# Comparing interspecific socio-communicative skills of socialized juvenile dogs and miniature pigs

**DOI:** 10.1007/s10071-019-01284-z

**Published:** 2019-06-29

**Authors:** Linda Gerencsér, Paula Pérez Fraga, Melinda Lovas, Dóra Újváry, Attila Andics

**Affiliations:** 1 Department of Ethology, Eötvös Loránd University, Budapest, Hungary; 2 MTA-ELTE ‘Lendület’ Neuroethology of Communication Research Group, Hungarian Academy of Sciences, Eötvös Loránd University, Pázmány P. s. 1/C, Budapest, 1117 Hungary

**Keywords:** Comparative, Dog, Pig, Interspecific communication, Human–animal interaction, Companion animal

## Abstract

Previous research proves dogs’ outstanding success in socio-communicative interactions with humans; however, little is known about other domestic species’ interspecific skills when kept as companion animals. Our aim was to assess highly socialized young miniature pigs’ spontaneous reactions in interactions with humans in direct comparison with that of young family dogs. All subjects experienced similar amount of socialization in human families. In Study 1, we investigated the appearance of human-oriented behaviours without the presence of food (Control condition) when a previously provided food reward was withheld (Food condition). In Study 2, we measured responsiveness to two types of the distal pointing gesture (dynamic sustained and momentary) in a two-way object choice test. In the Control condition of Study 1, the duration of pigs’ and dogs’ orientation towards and their frequency of touching the human’s body was similar. In the Food condition, these behaviours and orienting to the human’s face were intensified in both species. However, pigs exhibited face-orientation to an overall lesser extent and almost exclusively in the Food condition. In Study 2, only dogs relied spontaneously on the distal dynamic-sustained pointing gesture, while all pigs developed side bias. The results suggest that individual familiarization to a human environment enables the spontaneous appearance of similar socio-communicative behaviours in dogs and pigs, however, species predispositions might cause differences in the display of specific signals as well as in the success of spontaneously responding to certain types of the human pointing gestures.

**Electronic supplementary material:**

The online version of this article (10.1007/s10071-019-01284-z) contains supplementary material, which is available to authorized users.

## Introduction

Behavioural data from the literature provide evidence that domestic (e.g., cats, goats, and horses), as well as non-domestic (e.g., seals, monkeys, and great apes) animal species are able to successfully engage in interspecific communicative interactions with humans; more specifically, they seem to be responsive to human referential signals (for a review see Miklósi and Soproni 2006). The factors that shape individuals’ success in interspecific communicative interactions have been in the focus of recent interest and the extent of their defining roles is still under investigation. Differences in the performance of domestic animals and their wild relatives, as well as that of domestic and non-domestic species imply that along with species-specific ontogenetic effects, the emergence of these skills at least to some extent has been selected for during domestication (e.g., Miklósi et al. 2003; Hare and Tomasello 2005; Bräuer et al. 2006). In addition, learning by experience, i.e., socialization in a human environment might affect animals’ socio-communicative skills and thus their performance in such contexts (Barrera et al. 2011; Udell et al. 2010).

Dogs (*Canis familiaris*) and pigs (*Sus scrofa domesticus*) are both highly social, group-living domestic animals with domestication histories of clearly different duration and trajectories. The process of dogs’ domestication began considerably earlier (> 15,000 years ago, Thalmann et al. 2013) than that of pigs (< 10,000 years ago, Groenen et al. 2012). Nevertheless, the ancestors of both species must have been attracted to ancient human settlements by leftover food (Frantz et al. 2016), requiring similarly reduced fear and aggression together with increased tolerance of close human contact. Consequently, the feeding ecology of both species became characterized by human refuse scavenging, both became highly dependent on humans—as a food source—for survival. As opposed to the intensive usage of dogs for various work-related purposes throughout history (e.g., hunting, guarding and shepherding) and their current popular role as a companion animal living in and as part of the human family, pigs, on the other hand, have been used until very recently as a livestock animal species almost exclusively. Traits in dogs’ behaviour such as willingness to closely cooperate with humans must have been important criteria during their domestication process (Miklósi et al. 2003; Hare and Tomasello 2005; Gácsi et al. 2009a)—though whether this has played a major role has been questioned recently (Hare et al. 2010; Katz and Huber 2018), while excessive breeding and optimizing meat stock was the most important criteria for pigs.

Vast amount of behavioural data from the literature shows that family dogs are remarkably skilful in getting engaged in interspecific communicative interactions with humans, meaning that they do not only read diverse human signals, but readily display communicative behaviours themselves (Miklósi and Topál 2013; Katz and Huber 2018). They are able to flexibly use various forms of the pointing gesture to locate hidden food reward; they are successful in following proximal and distal pointing with arms, legs, even in complex situations (for a review, see Kaminski and Nitzschner 2013) or even following gaze direction (Met et al. 2014; Duranton et al. 2017). They also readily initiate and use eye contact as a form of social interaction when previously provided food reward is withheld (e.g., Gácsi et al. 2005; Bentosela et al. 2016) or inaccessible for them (e.g., Miklósi et al. 2003; Passalacqua et al. 2011), and they use also vocalization as an attention-getting behaviour (Miklósi et al. 2000). Dogs seem to be unique in a sense that—as contrary to their wolf ancestor—they have a strong species-specific preference for interacting with humans (Gácsi et al. 2005). Furthermore, dogs tend to exhibit the above-listed socio-communicative behaviours without any special training and already from a very early age on (e.g., Gácsi et al. 2005, 2009a; Riedel et al. 2008). Differences between dogs and their wolf ancestor related to the display of these behaviours show that dogs acquired their skills through the process of domestication (Hare et al. 2002; Kaminski and Nitzschner 2013). While dogs have a flexible behavioural system that is buffered against environmental effects (Gácsi et al. 2005), there is some evidence that former experience can also influence the appearance of their interspecific communicative behaviours in certain contexts (Udell et al. 2008; Barrera et al. 2011; Marshall-Pescini et al. 2008).

On the other hand, scientific interest to date had predominantly focused on the intraspecific social interactions of pigs kept together either under farm or laboratory conditions (Gieling et al. 2011; Marino and Colvin 2015). Although recent studies have already investigated several aspects of pig–human interactions as well (e.g., Tallet et al. 2010; Brajon et al. 2015; Bensoussan et al. 2019), the available knowledge about pigs’ interspecific social skills when interacting with humans is still limited as compared to what we know about dogs in this respect. Nawroth et al. (2013) have shown that young group-living farm pigs seem to react somewhat differentially to the different attentive states of a human (Nawroth et al. 2013). In spite of the fact that in one study farm pigs did not prove to be sensitive to human-given referential signals (i.e., pointing gesture) (Albiach-Serrano et al. 2012), with notable amount of previous training sessions farm-housed piglets were able to learn to use both proximal and distal pointing gestures to find hidden food reward (Nawroth et al. 2014, 2016; Bensoussan et al. 2016).

With the increasing recognition of the pig as a model species for biomedical research (Conrad et al. 2012; Helke et al. 2016), miniature pig breeds with small size (adult weight 30–60 kg) appeared (Gieling et al. 2011). Due to their reduced size and the sophisticated socio-cognitive capacities of pigs in general, the number of miniature pigs kept as companion animals has increased considerably in the last several years (Marino and Colvin 2015). When kept as companion animals, dogs and pigs occupy a similar ecological niche; they live among humans from an early age in a highly similar environment enriched in human social stimuli and contact.

While some data point towards similarities in dogs’ and pigs’ socio-cognitive skills—including the recognition of conspecifics or even humans, learning readily by reinforcement, or the fact that both species naturally rely on acoustic, olfactory, and visual cues in communicative interactions (for reviews see Gieling et al. 2011; Marino and Colvin 2015; Miklósi and Topál 2013; Miklósi 2015)—to the authors’ present knowledge, no former studies investigated highly socialized miniature pigs’ (i.e., kept as companion animals) interspecific social skills, neither on their own nor in direct comparison with that of family dogs. Given the similar living environments, comparable amount of exposure to complex social stimuli, situations, and the similar roles the two species occupy when kept as companion animals, by comparing their behaviour during human–animal interactions, we might learn valuable information about the contribution of species-specific traits and learning through experience to the development of interspecific socio-communicative skills (Miklósi et al. 2005). In addition, collecting data already at a young age is also important, since it allows for later follow-up comparisons to see whether and how specific behaviours might change throughout ontogeny.

Consequently, our aim was to assess young miniature pigs’ spontaneous reactions in interactions with humans in direct comparison with that of young dogs, both species experiencing similar amounts of social exposure to humans in human households; in two separate studies, we evaluated the animals’ readiness to display socio-communicative behaviours and to respond to human-given social cues. Both studies relied on paradigms that have already been well established for studying the interspecific social skills of dogs. In Study 1, we investigated the appearance of spontaneous human-oriented behaviours without the presence of food as well as when previously provided food reward was withheld. In Study 2, we were interested in the animals’ responsiveness to two different types of the human pointing gesture indicating hidden food reward in a two-way object choice test. We chose the feeding situation for testing, because humans feed their animals day by day, during which they supposedly behave similarly with both species, and thus, we consider that to be a fairly comparable context. Based on the similarities in their species-specific socio-communicative and learning abilities, as well as the fact that they have been able to successfully adapt to the human social environment, we hypothesized that the two species—given the similar rearing environments—communicate with humans and react to human cues in a similar manner.

## Ethical statement

Animal owners volunteered to participate in the studies, which were non-invasive, not causing any pain or suffering to the animals. We obtained a written official statement (#PE/EA/430–6/2018) from the Food Chain Safety and Animal Health Directorate Government Office about the decision of the Scientific Ethic Council of Animal Experiments. According to this statement and the corresponding definition by law in our country, the current non-invasive observational studies are not considered to be animal experiments. Based on this, we obtained the necessary permission from the University Institutional Animal Care and Use Committee as well (UIACUC, Eötvös Loránd University, Hungary).

## General methods

Both studies were carried out in the laboratory (4.45 m × 3.68 m room) of the Department of Ethology (Eötvös Loránd University, Hungary) in the presence of the animals’ owners and a female experimenter. None of the subjects showed any species-specific behaviours indicative of excessive fear or frustration (including aggression) throughout the experiments.

### Subjects

All animals enrolled in the studies were living in families exposed to similarly close human contact from their age of ~ 8 weeks. All subjects were mother-reared, living with their mother and littermates before weaning, where they were exposed to regular human contact. The pigs were from different litters with at least one parent different, and they were acquired from six different breeders in Hungary after all the necessary veterinary screening examinations. All the pigs are part of a long-term scientific project (https://etologia.elte.hu/en/lendulet-neuroethology-of-communication/) which required strict a priori selection procedure of the owners who volunteered for participation and close cooperation with the Department of Ethology for several years. The adoption process itself was supervised and guidelines were also provided for the piglets’ handling at home, socialization to humans, exposure and habituation to different environments, transportation, etc. to make the rearing environment as similar to that of a well-socialized family dog as possible. Most of the dogs’ owners were regular volunteers of the Family Dog Project (https://familydogproject.elte.hu/) and the socialization background of the dogs—as based on the owner’s report prior to enrolment—was similar to that of the pigs.

## Study 1

In this experiment, we aimed to investigate and compare the two species’ spontaneous behaviours exhibited towards an unfamiliar experimenter in two unrestricted consecutive short sessions, without the presence of food and when previously provided food reward was withheld. We partly based our method on the work done by Bensoussan et al. (2016).

### Methods

Our subjects were ~ 4-month-old juvenile family pigs (*N* = 10; 6 neutered males and 4 intact females; *X*_age_ ± SD = 4.2 ± 0.8 months; Minnesota and mixed miniature variants) and dogs (*N* = 10; 6 intact males and 4 females; *X*_age_ ± SD = 3.7 ± 1.0 months; from 8 different breeds) (see Online Resource 2 for detailed information about the subjects).

The test room was equipped with a table positioned next to the wall (holding a camera tripod and a plastic container with or without food), as well as a chair for the owner at 1.5 m distance from the table and equidistant from the two longer sides of the room. Before the test session began, the subject (S) was let free in the test room to explore the environment for 5 min, and its behaviour was not restricted in any way during the experimental procedure either. The owner (O) sat on a chair—at 2 m distance from the experimenter (E)—and remained passive throughout the experiment. We followed the method by Bensoussan et al. (2016), and we added a Control condition as well. The experiment consisted of two 120 s long sessions; the Control condition followed by the Food condition in fixed order after a short break, and both of them consisted of similar pre-test and test phases.

#### Control condition

During the *pre-test phase* ( ~ 1 min), E kneeled down and sat on her heels with her back against the wall while placing an empty plastic container on the table next to her, out of the animal’s reach, making sure that S follows the container’s path to the table. Then, each 20 s for five times E imitated the action of taking a treat out of the container and delivering it on the floor. By doing this, she also called the S’s name (several times if necessary) to make sure that S approaches her and has the chance to observe her action. The *test phase* followed the pre-test phase immediately: after imitating the delivery one last treat (the 6th one), E hid her hands behind her back and stayed passive (sitting on her heels) for the following 120 s. She followed S with her gaze and tried to establish eye contact.

#### Food condition

The E behaved the same way as described above in the Control condition, the only difference from the Control condition was the presence of food; during both the *pre-test* and *test phases*, E, instead of imitating the action, actually took treats out of the plastic container and delivered them on the floor. Treat delivery during the pre-test phase also served for testing the S’s motivation for eating the offered treat (small pieces of sausage for the dogs and dry dog food for the pigs, since based on pilot trials and owner reports these proved to be of similarly high value for the animals).

All animals approached E and followed her actions during both conditions’ pre-test phase and ate all offered treats immediately.

#### Behavioural analysis

All tests were video-recorded for later behavioural analysis using Solomon Coder; a program developed for coding behavior, where the user can define a set of behaviors/events, open and play back video files, record the behaviours into a coding sheet, and extract primary statistics, such as frequencies, durations, etc. (v. 090,913; © András Péter https://solomoncoder.com). We measured the following behaviours during the 120 s measured from the moment of the E hiding her hands behind her back and staying passive:

Body-orientation (duration, s): S is close to the E (within a max. distance of 30 cm) and orients its head towards any parts of her body (but not towards her face) while not touching the E.

Body-touch (duration, s): S establishes physical contact with the E (e.g., nosing, licking, pawing), but is not orienting its head towards her face (the corresponding frequency count of this variable was used in the analysis﻿, i.e. the number of times the subject initiated the behaviour and displayed it without stopping).

Face-orientation (duration, s): S is close to the E (within a max. distance of 30 cm) and orients its head towards her face (the corresponding frequency count of this variable was used in the analysis). Due to species-specific anatomical characteristics (e.g., relative smaller size﻿ of pigs’ eyes and their more lateral position), determining whether a pig establishes eye contact with a human is less straightforward than in﻿ the case of dogs. That is the reason why we chose to measure the face-oriented behaviour (i.e., the nose/snout of the animal being oriented towards the human’s face), which is straightforward in both species.

Orientation to E (duration, s): S is close to the E (within a max. distance of 30 cm) and its head is oriented towards her (any body parts, including her face and including touching her body as well; derived by merging the above three mutually exclusive variables).

Vocalization (duration, s): S is vocalizing.

E-oriented vocalization (duration, s): concurrence of Vocalization and Orientation to E.

20% of the recordings of both species was coded by two different coders for Body-orientation, Body-touch, and Face-orientation. The agreement between the two raters—calculated based on the raw coding sheets, where the three mutually exclusive variables are coupled together as one variable with several levels—was near perfect (Cohen’s Kappa, *ĸ* = 0.89, *P* < 0.001 for pigs and *ĸ* = 0.83, *P* < 0.001 for dogs). The vocalization durations could be determined obviously based on the sonograms belonging to the video recordings; thus, we did not calculate interrater agreement for that variable.

#### Data analysis

We used the R statistical environment (v. 3.5.0. R Development Core Team) for data analysis. The continuous variables did not follow normal distributions, as indicated by Shapiro–Wilk tests. Using square root transformation, the variable Body-orientation fulfilled the assumptions of normality. For Face-orientation and Body-touch, a similar transformation did not result in normal distributions, so we used the corresponding frequency-count unit (that followed a Poisson distribution) during the analysis. Note that the correlation between the two variables of different units measuring the same behaviour proved to be strong (Spearman’s rank correlation, *r*_s_ = 0.903, *P* = 1.63 × 10^–15^ for Body-touch and *r*_s_ = 0.871, *P* = 2.84 × 10^–13^ for Face-orientation). For the transformed variable, we built a Linear Mixed-effects Model (LMM) fit by residual maximum likelihood (REML) and used Satterthwaite approximation for estimating the degrees of freedom. For the variables, Face-orientation and Body-touch (Poisson-distributed count data), we built Generalized Linear Mixed-effects Models (GLMMs) fit by maximum likelihood using Laplace Approximation. In each model, we included as fixed factors ‘Species’ (between-subject factor), ‘Condition’ (within-subject factor) and the interaction of these two factors, and individual subjects as a random factor. We obtained pairwise post-hoc comparisons for the fixed factors. Since four of the dog subjects did not produce any vocalizations in neither conditions and additional two of them vocalized only in one condition for a max. of 1 s duration, we analysed the vocalizations of the pigs only; we used Wilcoxon signed-rank tests for comparing the duration of Vocalization between the two conditions. To see whether there was any difference in the relative duration of pigs’ E-oriented vocalizations, we calculated the ratios of E-oriented vocalization and Orientation to E for both conditions and compared these values by Wilcoxon signed-rank tests. The data sets generated and analysed during the current study are available in the form of electronic supplementary material (Online Resource 2).

## Results

Detailed results of the LMM, GLMMs, and post-hoc tests are shown in Online Resource 1.

**Fig. 1:**
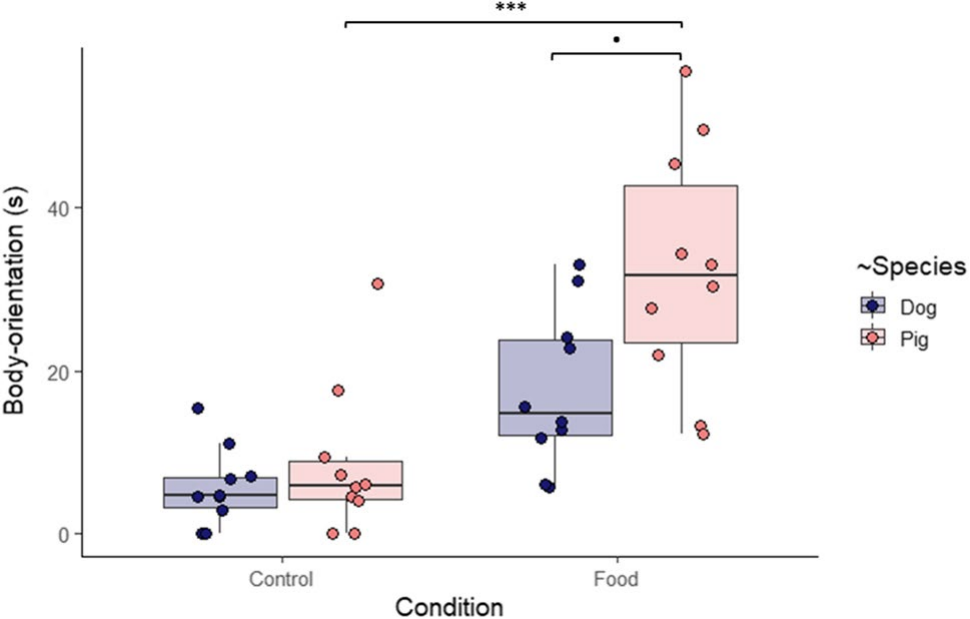
Duration of pigs’ and dogs’ orientation to the experimenter’s body in the Control and Food conditions. Bold lines stand for the median, boxes indicate the interquartile range and whiskers extend until the smallest and largest values (excluding outliers and extremities). The dots represent the individual data points. Significance codes: '***' < 0.001; '.' < 0.1 (see Online Resource 1)

‘Condition’ had a significant effect on the animals’ Body-orientation (*T* = 2.419, *P* = 0.026) and the interaction effect between ‘Species’ and ‘Condition’ was marginal (*T* = 1.970, *P* = 0.064) (LMM, model statistics: *X*^2^_3_ = 26.605, *N* = 20, *P* = 7.124 × 10^–6^). Pigs in the Food condition oriented more to the E’s body than in the Control condition and they also tended to orient more than dogs in the Food condition, whereas post-hoc comparisons revealed no difference between dogs’ Body-orientation in the two conditions (Fig. 1).

**Fig. 2:**
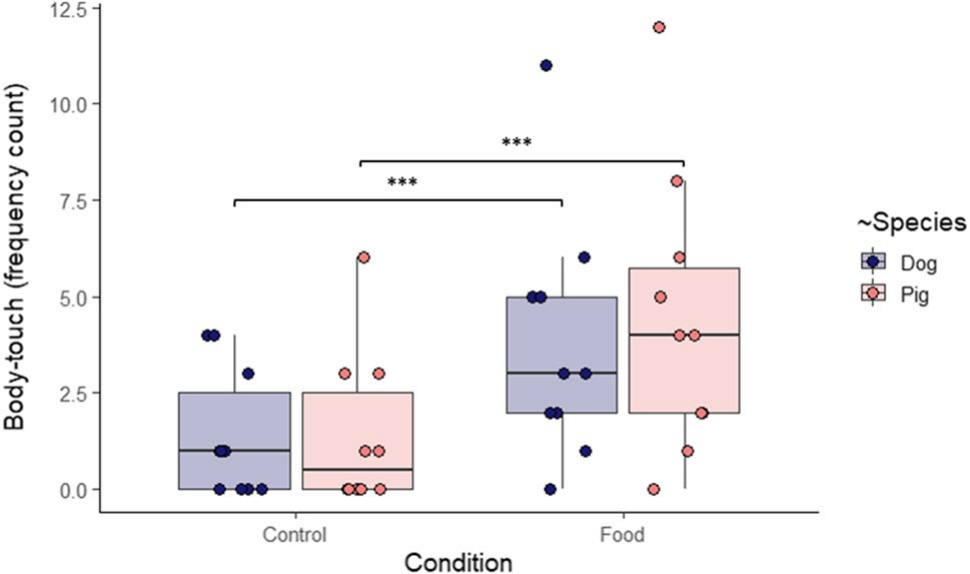
Frequency of pigs’ and dogs’ touching the experimenter’s body in the Control and Food conditions. Bold lines stand for the median, boxes indicate the interquartile range, and whiskers extend until the smallest and largest values (excluding outliers and extremities). The dots represent the individual data points. Significance code: '***' < 0.001 (see Online Resource 1)

‘Condition’ had a significant effect on Body-touch frequency (*Z* = 3.263, *P* = 0.001) (GLMM, model statistics: *X*^2^_3_ = 94.126, *N* = 20, *P* = 2.2 × 10^–16^). Both pigs and dogs touched the E more frequently in the Food than in the Control condition (Fig. 2).

**Fig. 3:**
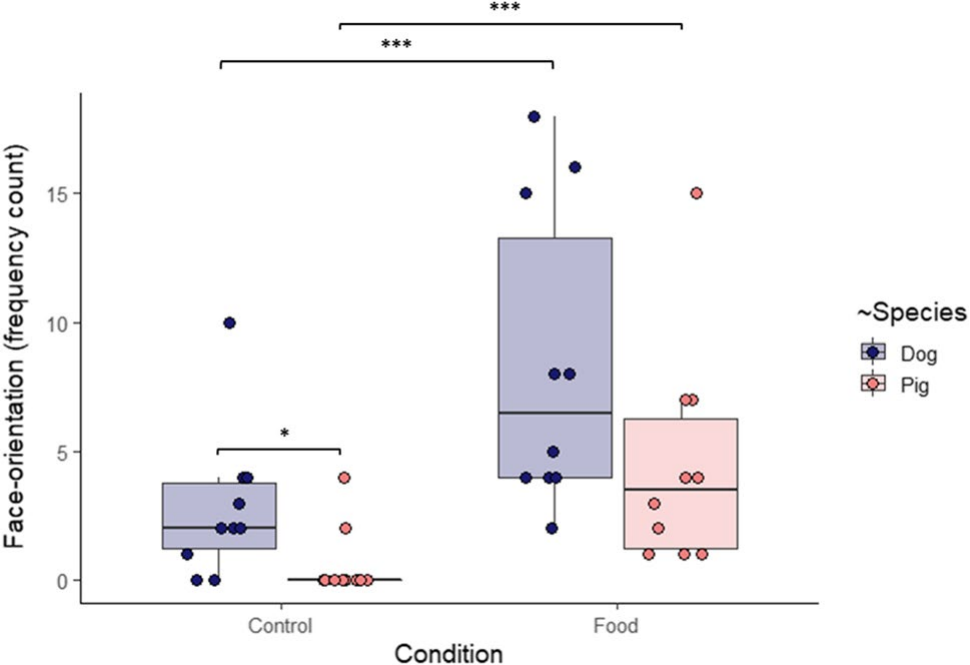
Frequency of pigs’ and dogs’ orientation to the experimenter’s face in the Control and Food conditions. Bold lines stand for the median, boxes indicate the interquartile range, and whiskers extend until the smallest and largest values (excluding outliers and extremities). The dots represent the individual data points. Significance codes: '***' < 0.001; '*' < 0.05 (see Online Resource 1)

Both ‘Species’ and ‘Condition’ affected significantly Face-orientation frequency (*Z* = − 3.083, *P* = 0.002 and *Z* = 5.1, *P* < 0.0001, respectively) and the interaction effect between the two factors was marginal (*Z* = 1.915, *P* = 0.055) (GLMM, model statistics: *X*^2^_3_ = 69.378, *N* = 20 *P* = 5.810 × 10^–15^). Both species looked more frequently to the E’s face in the Food than in the Control condition, and in the Control condition dogs looked more frequently than pigs (Fig. 3).

Neither the Vocalization (Wilcoxon signed-ranks test: *W* = 24, *N* = 10, *P* = 0.769), nor the relative duration of E-oriented vocalization (Wilcoxon signed-ranks test: *W* = 16, *N* = 10, *P* = 0.477) of pigs differed between the Control and Food conditions.

To sum up, our findings revealed similarities between pigs and dogs in their duration of orienting towards and the frequency of touching the experimenter’s body when no food reward had been provided before (Control condition). We also found that in the presence of food (Food condition), these behaviours, as well as the orientation to the experimenter’s face intensified in both species. However, pigs exhibited face-orientation to an overall lesser extent and almost exclusively in the Food condition.

## Study 2

We aimed to examine juvenile family pigs’ spontaneous sensitivity to human pointing gestures in a two-way object choice paradigm and to compare it to that of juvenile family dogs. As a human cue, we applied two forms of the distal pointing differing in their temporal parameters (i.e., dynamic sustained and momentary). Due to the increased distance between the target and the pointing hand, the distal pointing gesture has been found to be more demanding for several species than the proximal one, especially its momentary version when the gesture is not visible any more when the choice is made (see Miklósi and Soproni 2006 for a review). Thus, both spatial and temporal forms of the pointing gesture seem to be determining factors, and because of the above reasons, the momentary distal version has been established as a benchmark by the previous studies (Gácsi et al. 2009b).

### Methods

Our subjects were juvenile family pigs (*N* = 9; 5 neutered males and 4 intact females, *X*_age_ ± SD = 6.1 ± 1.7 months; Minnesota and mixed miniature variants) and family dogs (*N* = 9; 5 intact males and 4 intact females; *X*_age_ ± SD = 5.9 ± 1.9 months; 7 different breeds and 2 mixed breeds) (see Online Resource 2 for detailed information about the subjects). All the pigs had participated in Study 1 previously, while the dogs were a new group of randomly selected subjects. One pig had to be excluded from the test during the familiarization phase (see below) of the procedure, because she did not bear being restrained and thus was not able to pay attention to the E, so we used the data of *N* = 8 pigs in the analysis.

The apparatus consisted of two identical, opaque red plastic containers (13.5 × 13.5 × 5.5 cm) that were double-bottomed, both containing pieces of unreachable food to control for odor cues.

#### Familiarization phase

**Fig. 4:**
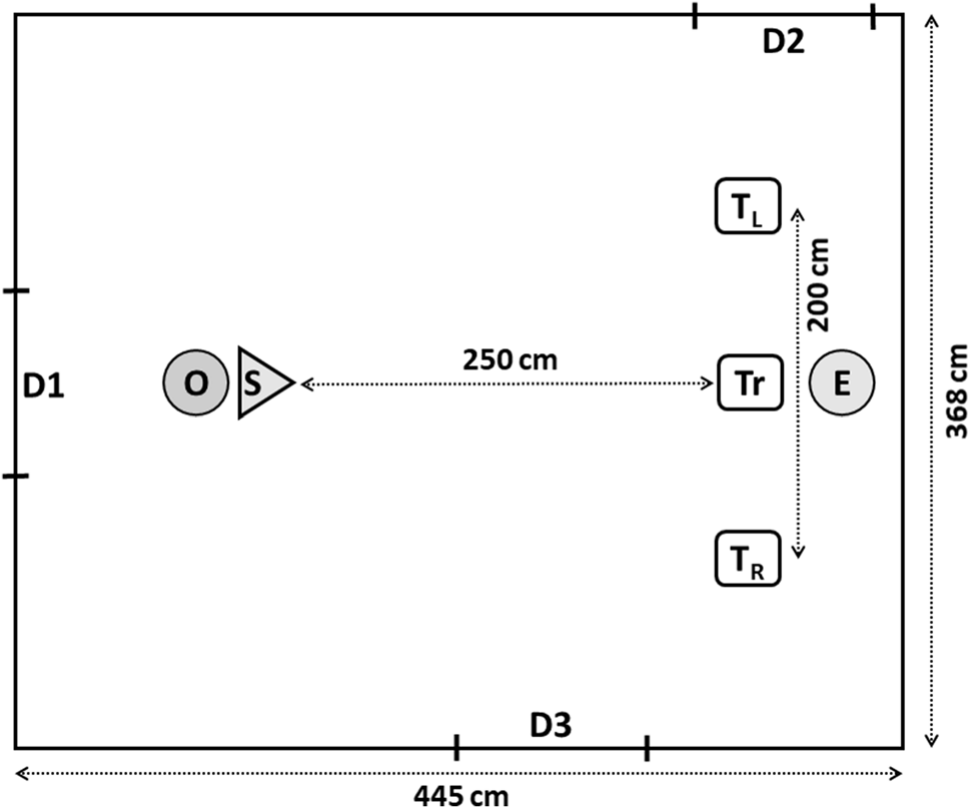
Experimental setup for Study 2. ‘O’ marks the position of the owner, ‘S’ marks that of the subject and ‘E’ marks that of the experimenter during the training and test trials. ‘Tr’ shows the position of the container in the training trials, while the ‘T’s show the position of the two containers in the test trials, letters in lower index mark the left (L) and right (R) sides from the subjects’ point of view. D1, D2 and D3 indicate doors (of which D1 was used during the experiment)

After entering the test room, the subject (S) was allowed to explore for 5 min in the presence of the owner (O) and the experimenter (E). At the end of the familiarization phase—to make sure that the animal was motivated to eat the food reward from the plastic containers—the O sat down on the floor making hold of the S, while the E placed one container to the floor in front of the S approx. 50 cm away, showed a piece of food (small pieces of sausage for the dogs and dry dog food for the pigs), placed it in the container and the animal was released to eat the food. This was done once with both containers. Then, 2–3 *training trials* followed to make the animals familiar with the task and the test situation, and to make sure that they were motivated enough to go for the food on subsequent trials. The O sat down at a predetermined point holding the S by its harness or its body. The E placed one container 2.5 m away from them and kneeled down facing the S at a position of approx. 30 cm behind the container (Fig. 4). She placed a piece of food in the container in full view of the subject, and then, the O let the animal free to approach and eat the food. This was repeated at least once with each container. The criterion was to approach the container immediately and eat the food twice in a row within a maximum of three trials.

#### Test trials

The *test trials* immediately followed the training trials. O was holding the animal and E was at a kneeling position facing them at approx. 2.8 m distance. Before the trials, the kneeling E was holding both containers in front of her body and placed one piece of food into one of them, kept exchanging them in her hands two times (to prevent S from knowing, where the food was) and then placed them on the floor 2.5 m away from S, 2 m apart from each other, one after the other always in the same order, with the order being balanced across subjects. E was positioned equidistant from the two containers and approx. 30 cm behind the midline between them (Fig. 4). Before the pointing gesture, E with her arms bent in front of her chest gave an attention-getting signal (making short, clapping sounds with her mouth) and tried to establish eye-contact with S. The pointing gestures immediately followed the attention-getting signal and were presented when S was standing still facing E.

The subjects were presented with two conditions differing in the type of the pointing gesture, which were distal dynamic sustained (DDS) and distal momentary (DM) pointing (based on Miklósi and Soproni 2006). In the DDS condition, E was pointing with one extended arm towards the baited container while looking at S until S made its choice. In the DM condition, the same pointing lasted for only about 1 s, and then, E pulled her arm back to her chest and kept looking at S. The distance between the tip of the index finger and the container was approx. 70 cm in both conditions. S was released to choose immediately after the pointing gesture. If S did not leave the start position in 5 s, E repeated the same gesture once more (together with the attention-getting signal). The container first approached by S within 5 cm was considered as chosen. After choosing the baited container, S was allowed to eat the food and was praised verbally by its owner. After S made its choice, the experimenter quickly picked up both containers; thus, if S chose the empty container first, then he/she failed to get the food. If the animal stopped choosing any of the containers during the test, then one training trial was introduced. The subjects were presented with 28 trials altogether (14 trials/condition: 7–7 trials to both sides) within three sessions (10–10–8), interrupted by two 5 min breaks.

After each break and before the new session, one training trial was introduced. Reward side and gesture type were semi-randomized and the first trial was counterbalanced across subjects with no side and gesture type being presented more than two times in a row, and the first two trials were always different.

#### Control trials

To find out more about the robustness of pigs’ performance (see “Results”) on a different occasion (within a one week interval), 14 control trials (i.e., post-test trials with no human cueing) were run with them in the same experimental setup with 2 training trials before the actual trials. The control trials differed from the other test trials in a way that E did not produce any gestures to indicate the baited container. She was kneeling with her arms resting besides her body looking straight at S after placing the two bowls on the floor, and S was allowed to choose from the two containers in approx. 2 s.

#### Behavioural and data analysis

All sessions were videotaped and later coded. We measured the number of correct choices/conditions. Interrater reliability was not assessed for this variable, because the subjects’ choices could be determined without ambiguity. We used the R statistical environment (v. 3.5.0. R Development Core Team) for data analysis. To check for the effects of the two conditions and species on performance (i.e., number of correct choices), we used a two-factor mixed ANOVA (with within-subject factor ‘Condition’ and between-subject factor ‘Species’). We analyzed group-level performance in both conditions by one sample *t* tests, and used binomial tests to evaluate individual performance in both test conditions and also to identify any side bias (for conditions: at least 11/14 correct choice/condition; *P* < 0.03, and for side bias: choosing the same side at least 19/28 times during the first test session; *P* < 0.05 and at least 11/14 choices to the same side for pigs during the control trials; *P* < 0.03). To evaluate the consistency of pigs’ side bias performance on the two occasions, we calculated for each individual the proportion of choices to the biased side in both the test and control trials and tested the correlation between the two proportions by Spearman’s rank correlation. The data sets generated and analysed during the current study are available in the form of electronic supplementary material (Online Resource 2).

### Results

**Fig. 5:**
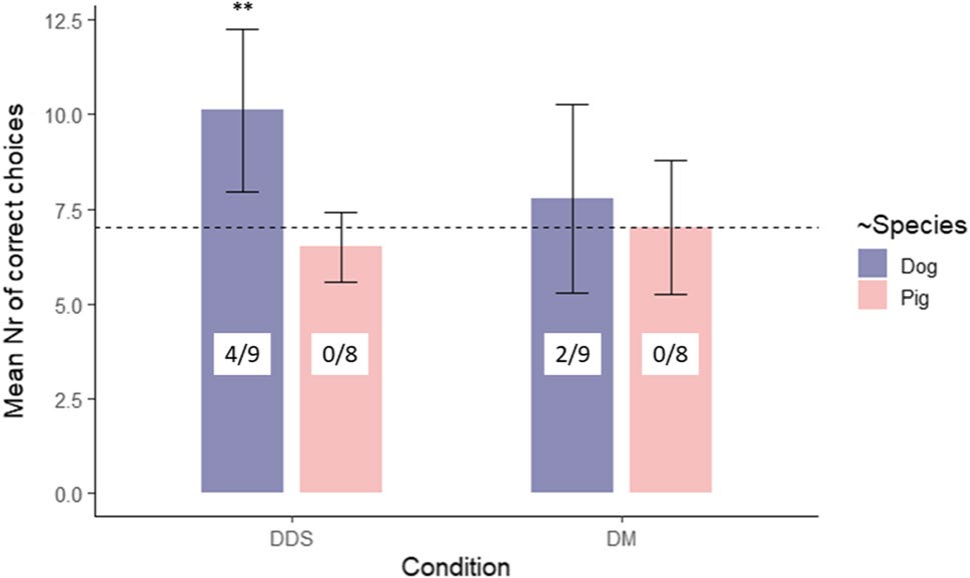
Mean number of correct choices of dogs and pigs in the two conditions. Numbers in the bars indicate the amount of subjects performing above chance on individual-level versus the total number of subjects. *DDS* distal dynamic-sustained pointing, *DM* distal momentary pointing. The dashed line represents the chance level, the error bars show standard deviations. Significance code: '**' < 0.01 (against the chance level)

We found an interaction effect between ‘Condition’ and ‘Species’ (ANOVA, *F*_1,15_ = 9.808, *P* = 0.007) on the number of correct choices. Further group-level analysis confirmed that dogs as a group performed above the chance level in the DDS condition (one sample *t* test, DSS_dog_: *t*_8_ = 4.346, *P* = 0.002), while their performance in the DM condition and pigs’ performance in both conditions did not differ significantly from chance (one sample *t* tests, DM_dog_: *t*_8_ = 0.938, *P* = 0.376; DDS_pig_: *t*_7_ = − 1.528, *P* = 0.17; DM_pig_: *t*_7_ = 0 *P* = 1, Fig. 5). Individual performance analysis revealed that no pigs were above the chance level in both conditions. Among the dogs 4/9 were above the chance level in the DDS condition and 2/9 were above the chance level in the DM condition.

**Fig. 6:**
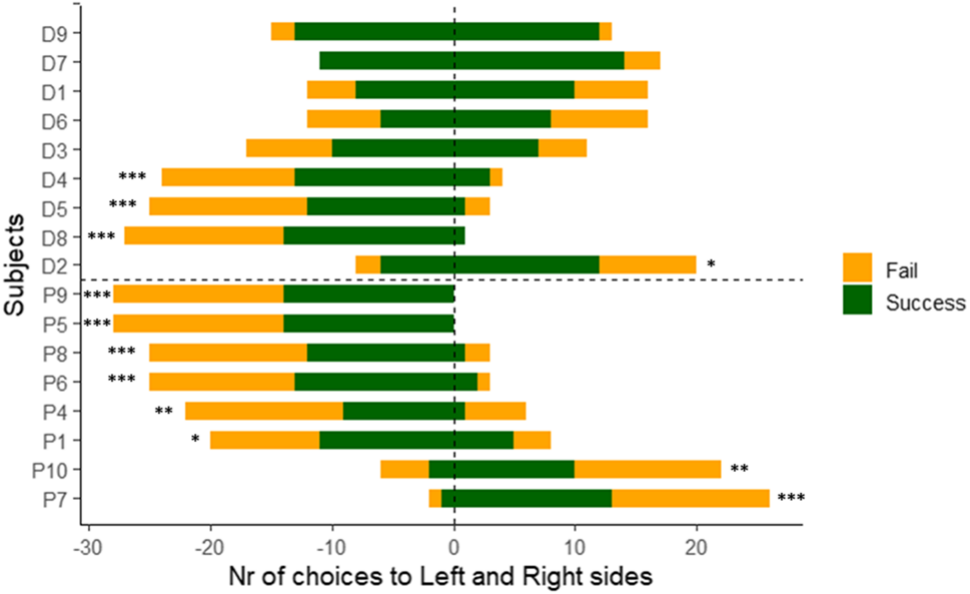
Number of individual choices to left and right sides in the test trials. The y axis represents individual subjects; *D* dog, *P* pig. Negative values on the x axis show choices to the left, while positive values show choices to the right side (from the subject’s point of view, see also Fig. 4). ‘Fail’ stands for incorrect and ‘Success’ stands for correct choices. Significance codes (for side bias, Binomial tests): '***' < 0.001; '**' < 0.01; '*' < 0.05

Concerning the performance on the first trial only, 6/8 pigs went to the left and 6/9 dogs to the right side, neither of which can be regarded as a species-specific lateralization effect (Binomial tests, *P* = 0.145 for pigs and *P* = 0.254 for dogs). Further individual-level analysis of all trials showed that all the pigs and some dogs exhibited a side bias either to the left (*N*_pigs_ = 6/8, *N*_dogs_ = 3/9) or to the right (*N*_pigs_ = 2/8, *N*_dogs_ = 1/9) side (Fig. 6), and the side bias did not depend on the placement order of the two containers (Fisher’s exact test, *P* = 1). 4/8 pigs and 2/4 dogs developed overall bias to the side to which they went first—the ratios are the same in the case of both species whether we consider first choice irrespective of success, or first successful choice. This means that the side bias did not depend on the animals’ first (or first successful) choice either (Fisher’s exact test, *P* = 1) (see Online resource 2 for the ratio of subjects per trial choosing the same side as the later preferred/biased). During the control trials, pigs’ group performance was again on chance level (one sample *t* test, *t*_7_ = − 0.886, *P* = 0.405), but as opposed to the first occasion, only 5/8 pigs showed side bias, all of them to the same side as previously (*N* = 4/8 to the left and *N* = 1/8 to the right side). In line with this, we found positive correlation between the proportion of choices to the biased side in the test and control trials (Spearman's rank correlation: *r*_s_ = 0.89, *N* = 8, *P* = 0.003, see Fig. S1 in Online Resource 1).

To sum up, in the present two-way choice task, neither of the two species followed the distal momentary pointing gesture above the chance level, and only dogs relied spontaneously on the distal dynamic-sustained version of the same gesture. Regardless of gesture type, all pigs—while only less than half of the dogs—developed a clear lateralized behaviour (i.e., overall side bias), the direction of which did not depend on the individuals’ first (successful) choice.

## General discussion

To the authors’ present knowledge, our work is the first one presenting similarities and differences in juvenile family dogs’ and miniature family pigs’ behaviour during interspecific interactions with humans. As found in our first study, pigs show some similar patterns of spontaneous human-oriented behaviours as family dogs in an unrestricted neutral context without the presence of food (Control condition), as reflected by the time spent with orienting towards the human’s body in close proximity and also the frequency of establishing physical contact with the human. The experience of being fed by the human and the presence of food (Food condition) intensify these behaviours, as well as orienting to the human’s face in both species. However, the presence of food seems to affect pigs’ orientation to the human’s body to a greater extent than that of dogs. Furthermore, our results also point out that the experience of being fed by the human plays a major role in triggering the display of face-orientation only in pigs but not in dogs. Most dogs tended to orient voluntarily at the unfamiliar human partner’s face even when they had not been provided any food right before, while the majority of the pig subjects did not perform any face-orientation during the Control condition and they exhibited this behaviour to an overall lesser extent. In our second study, only dogs but not pigs showed evidence of spontaneously relying on a human distal dynamic-sustained pointing as directional signal for locating a hidden food reward in a two-way object choice test. As a group, neither of the two species was successful in following the momentary version of the pointing gesture, whereas all pigs (while only some of the dogs) showed a clear lateralized behaviour (i.e., overall side bias).

The found similarities in the two species’ behaviour towards the unfamiliar human partner in Study 1, such as approaching her voluntarily within close proximity, touching her body, etc. let us infer that the socialization background of our subjects in terms of making the presence of humans in general safe and comfortable can be regarded as comparable. This allows for ruling out that any emerging differences should be due to pigs being generally more fearful of the situations and/or the experimenter than dogs. The observation that both species were performing a considerable amount of explorative behaviour in the Control condition—besides the explicitly coded ones—gives further support to the above argument. All this is important, since multiple works with farm pigs pointed out the appearance of fearful or aggressive behaviours at the onset of the experimental procedures that made it necessary to include several occasions of familiarization procedures before the start of the data collection (e.g., Bensoussan et al. 2016; Nawroth et al. 2013, 2014). Therefore, working with family pigs instead of farm animals supports the comparability of the two species’ behaviour in the present experimental procedures.

Besides the similarities in young dogs’ and pigs’ spontaneously exhibited human-oriented behaviours, our findings imply an important difference between the two species in their readiness to orient at the human partner’s face. This behaviour (and more specifically the establishment of eye contact) is widely reported in dogs; it typically appears spontaneously without special training and is regarded as an interspecific communication skill that domestication seems to have uniquely strengthened in them (e.g., Hare et al. 2002; Miklósi et al. 2003). An earlier study comparing dogs’ human-oriented gazing with that of another domestic species found that dogs gazed earlier and for longer duration at the human (not specifying body parts) than cats did in a problem solving situation in a feeding context (Miklósi et al. 2005). The authors explained the found differences in terms of the two species’ different levels of independence from humans, which might as well be rooted in the differential selection pressures during the course of domestication. Such evolutionary differences hold true for dogs and pigs as well. Pigs were not selected for working in close cooperation with humans that would have made it important for them to seek information about human attentional states, which might explain—on an evolutionary level—to some extent the species’ overall lower tendency in displaying specifically the face-oriented behaviour. Along with these, it is also important to note here that all experimenter-oriented behaviours were scored from within a max. distance of 30 cm from the experimenter (because of practical reasons). Due to anatomical differences between the two species, however, it might be more difficult for a pig than for a dog to raise the head at the necessary angle to perform face-orientation for a longer period (while body-orientation can be performed even with a more neutral head position). Consequently, we cannot rule out the possible influence of this anatomical factor on the above outlined findings.

Interestingly, the above does not apply if we consider orienting towards the human’s body. Pigs and dogs exhibited Body-orientation for a similar duration in the Control condition, while pigs tended to orient more towards the human’s body than dogs in the Food condition. One possible explanation to the fact that the presence of food intensified pigs’ body-oriented behaviour to a greater extent than that of dogs might lie in the difference between the two species’ motivational states to receive the expected food from the human. The two species might also differ in their persistence to anticipate food that they had just experienced to get, which might as well relate to general motivational differences.

Looking at the functional perspective, the clear appearance of pigs’ face-orientation in the Food condition only, along with the increase in that of dogs and the increased body-orientation and body-touching of both species in the Food condition might indicate the communicative, attention-getting nature of these behaviours not only in dogs, but also in pigs. Furthermore, considering the apparent intensification of all the measured experimenter-oriented behaviours in the presence of food, we can assume an underlying role of simple associative learning mechanisms, i.e., learning through former daily routines about food coming from the human, which all subjects must have experienced. Nawroth et al. (2013) reported on the tendency of juvenile farm pigs for discriminating two humans based on their attentive states (i.e., head orientation) after some training (Nawroth et al. 2013), while adult family dogs were reported to have the ability of recognizing human attention without any training introduced specifically for such purpose (Gácsi et al. 2004). These can be related to our finding that young pigs display face orientation almost exclusively in the feeding context, which also suggests the necessity of previous learning processes for this behaviour to appear apparently in this species, whereas it seems to be displayed more unconditionally in young dogs.

In general, all pigs vocalized during Study 1, while only a few dogs did. The vast majority of pigs’ vocalizations during both short sessions can be best characterized by—without aiming here for precise classification based on acoustic parameters—the general “grunt” label (Tallet et al. 2013). While pigs are highly vocal species and have been reported to produce diverse call types across different situations (e.g., Tallet et al. 2013; Linhart et al. 2015; for review, see Marino and Colvin 2015), a “grunt” is not a situation-specific call type and it might be produced across a wide range of social and non-social contexts (Linhart et al. 2015). Although recent studies have identified qualitative differences in grunts across different arousal states (Linhart et al. 2015) or emotional valence (Briefer et al. 2019), qualitative analysis based on acoustic parameters was beyond the scope of the present study, and quantitative results do not provide evidence neither for the influence of the context, nor for the interspecific communicative nature of pigs’ calls in the present experimental setup.

Along with all the above, we found important differences in the two species’ readiness in spontaneously adjusting their behaviour according to the visual communicative signals of a human in a two-way choice task. Although pigs’ visual acuity is known to be poorer than that of humans and dogs (Zonderland et al. 2008), we know that pigs can be trained to follow even the distal human pointing gesture in a comparable setting to ours (Nawroth et al. 2014), which shows that they possess the sensory abilities as well as the cognitive capacities that are necessary to fulfil the task. This indicates that the main difference between dogs and pigs in this sense is probably not in their cognitive capacity of learning human communicative cues. Since humans have proven to act as naturally salient social stimuli for dogs (e.g., Gácsi et al. 2005), this might serve as a facilitating mechanism in learning about human behaviour even without specific training. In contrast, there are no data indicating that the same would also hold true for pigs, and the different selection pressures during the two species’ domestication do not imply that either. Along with this, we cannot rule out that further social experience with humans would enable pigs to perform successfully in the same task at an older age, although former comparisons of dogs’ and wolves’ performances revealed that in some cases, even extensive socialization with humans does not necessarily help overcome natural species differences (Miklósi and Soproni 2006).

Interestingly, the side bias that emerged in 100% of the pig subjects during the test trials disappeared in three individuals during the following control session, while for the rest of the subjects, it was consistent in time (i.e., they retained the bias to the same side as previously). There is growing literature on lateralization including both population-level responses to certain stimuli (e.g., Siniscalchi et al. 2013; Andics et al. 2016) and individual-level motor lateralization (Tomkins, Thomson, and McGreevy 2010). Lateralized behaviour is commonly reported for choice paradigms as well, not only in farm animals (e.g., Kaminski et al. 2005; Nawroth et al. 2014), but a proportion of dog subjects, in two-way choice experiments, are also often reported to develop a preference to one side (e.g., Gácsi et al. 2009b; Prato-Previde et al. 2008). This decision-making rule—other than following the human cue—can also be considered a cost-efficient strategy yielding 50% success rate, and we might suppose that it is followed when the task itself is too difficult for the subject. There is evidence from the literature that pigs are able to use their spatial memory flexibly; they can be trained to either return to a location, where they previously found food (“win-stay” task) or to use the memory of a previously discovered food site to forage elsewhere (“win-shift” task), and they seemed to be more successful in the latter task (Marino and Colvin 2015). However, our findings—in accordance with other reports (Nawroth et al. 2014)—point out that in a two-way choice task, young pigs’ spontaneous overall performance pattern is rather similar to a “win-stay” strategy. Moreover, their tendency to develop preference for one side is quite robust; seems to be mostly consistent in time and context. For pigs, foraging is a natural species-specific activity, during which they heavily rely on spatial cues (Marino and Colvin 2015). Thus, they might have a stronger general inclination to use spatial cues than dogs, which might as well account for the found differences between the two species with regard to their predispositions to develop lateralized behaviour.

Apart from showing bias to one side, most of the pigs (6/8) made successful choices to both sides throughout the course of Study 2 (Fig. 6). This means that 75% of the pig subjects found food reward on both sides, so the lack of experience about either of the two sides being rewarded could possibly explain the choice pattern of those two individuals that only chose the same side. Also, we found no evidence for the location of the first successful choice influencing the side to which the subjects eventually developed a preference. During the initial training trials, we deliberately avoided baiting the actual test locations to exclude the possibility of biasing the subjects’ choice in the first test trial to the last rewarded side in the training trial. Along with these we cannot rule out that introducing the baited locations already in the training trials might have led to a potential better overall test performance for pigs.

Dogs’ performance in the distal dynamic-sustained pointing trials is in line with other data available in the literature (Kaminski and Nitzschner 2013). As opposed to several previous findings, however, dogs as a group were not successful in DM trials, although individual-level analysis revealed that two subjects’ performance was above chance level in this condition, and other studies with young dogs also reported that, in spite of the success on group level, less than half of the individuals performed above chance (e.g., Gácsi, Kara, et al. 2009a). The exact experimental setup and procedure details were also found to affect performance in the pointing task (Pongrácz et al. 2013). Consequently, slight differences in our procedure compared to others’ (e.g., lack of pre-training to both sides, as, e.g., in Gácsi et al. 2009b or Virányi et al. 2008) or the setup, such as somewhat bigger distance between the two objects and/or the tip of the pointing finger and the container (as in, e.g., Virányi et al. 2008) could also cause the task to be more difficult for the dogs participating in our study.

### Limitations

One particular advantage of this study, in line with some previous ones comparing, e.g., the interspecific social skills of dogs and cats (Miklósi et al. 2005) or wolves (e.g., Virányi et al. 2008) is that we aimed to ensure that subjects are raised in similar environments providing comparable social stimulation by humans. This reduces the chance that any differences that emerged are due to a general determinative difference in the two species’ experience with humans. Nevertheless, we have to keep in mind that we do not have exact information for either species about the extent to which the owners reinforced any of those behaviours during socialization that we specifically tested for in our two studies (e.g., establishment of eye-contact/face-to-face orientation or following any version of the pointing gesture). Almost all pig owners kept a dog as well as a pig, and a general request for them upon the pigs’ arrival was to treat the pig in a similar manner as they would treat a dog, as much as possible. In spite of this, we cannot rule out the possibility that the owners still behaved differently in general with pigs and dogs (i.e., having different overall attitudes, expectations, the quality of social bonding may be different, etc.), which in turn could affect daily learning and, therefore, have potential influence on subjects’ test performance.

The supervised socialization of the piglets, as well as the a priori selection of the owners to fit the strict enrolment criteria proved to be demanding and time consuming tasks. Due to this we had the opportunity to work with only a finite amount of subjects, which we need to take into account when evaluating our results. Furthermore, the peculiarity of the pig population, as well as the fact that most of the pig subjects belonged to the same Minnesota miniature breed (see Online resource 2 for details) also makes the generalization of the results in this sense limited. Considering the dog subjects, we tried to enrol a diversity of breeds in both studies (see Online resource 2 for details), since there is evidence that breed group could potentially affect performance in interspecific communicative tasks (e.g., Gácsi et al. 2009b; Passalacqua et al. 2011).

## Conclusion

To conclude, we used a comparative framework to provide evidence for the spontaneous emergence of similar socio-communicative behaviours in juvenile dogs and pigs—given intensive individual familiarization to human environment. To what extent these similarities are the result of learning by experience or rather due to similar species-specific predispositions needs further investigations. Consistent with data from the literature, our findings also suggest that species-predispositions can cause differences in the display of specific signals (such as orientation to human face), as well as in the success of spontaneously following certain types of visual communicative cues (distal pointing gestures) of a human. As one possible explanation of our findings, we infer that dogs and pigs do not differ essentially in their cognitive capacity of learning to follow interspecific communicative cues, but the natural salience of the human as social stimulus for dogs might facilitate such learning to take place without specific training. Our results are also informative with regard to the miniature pig as a new candidate model for studying human-animal interactions, and the potentials of the species’ usage in comparative ethological research.

## Electronic supplementary material

Below is the link to the electronic supplementary material.

Supplementary file1 (XLSX 21 kb)

Supplementary file2 (PDF 191 kb)
