# Supplementary material for: Comparing interspecific socio-communicative skills of socialized juvenile dogs and miniature pigs
Source: Anim Cogn. 2019 Jun 29;22(6):917–29. doi: 10.1007/s10071-019-01284-z (PMC6834752; doi:10.1007/s10071-019-01284-z)
Supplement: Supplementary file 2 — Supplementary file2 (PDF 191 kb) [file 10071_2019_1284_MOESM2_ESM.pdf]

Supplementary Information for

Comparing interspecific socio-communicative skills of socialized juvenile dogs and miniature pigs

Authors and affiliations

Linda Gerencsér[1,2*], Paula Perez Fraga[1,2], Melinda Lovas[1], Dóra Ujváry[1], Attila Andics[1,2]

[1] Department of Ethology, Eötvös Loránd University, Budapest, Hungary

[2] MTA-ELTE 'Lendület' Neuroethology of Communication Research Group, Hungarian Academy of Sciences – Eötvös Loránd University, Budapest, Hungary

*Correspondence: linda.gerencser@gmail.com
ORCID ID: https://orcid.org/0000-0001-7030-178X
Tel: +36 1 381 2179
Address: Department of Ethology, Eötvös Loránd University, 1117 Budapest, Pázmány P. s. 1/C, Hungary

Supplementary Tables

**Fixed effects**	**Estimate**	**Std. Error**	**df**	***t* value**	***P* value**
Species	-0.069	0.567	35.856	-0.123	0.903
Condition	1.328	0.549	18	2.419	0.026 *
Species * Condition	1.529	0.776	18	1.97	0.064 .

Note. Significance codes: '***' < 0.001; '**' < 0.01; '*' < 0.05; '.' < 0.1.

Table S1 *P* values and related parameters for the main effects and their interaction on Body-orientation (LMM)

**Contrast**	**Estimate**	**SE**	**df**	**t ratio**	***P* value**
Dog, Control - Pig, Control	0.069	0.567	35.86	0.123	0.999
Dog, Control - Dog, Food	-1.328	0.549	18	-2.419	0.109
Pig, Control - Pig, Food	-2.858	0.549	18	-5.206	0.0003 ***
Dog, Food - Pig, Food	-1.460	0.567	35.86	-2.574	0.0654 .

Note. Significance codes: '***' < 0.001; '**' < 0.01; '*' < 0.05; '.' < 0.1. Tukey method for *P* value adjustment.

Table S2. *P* values and related parameters of the post hoc tests for the contrasts on Body-orientation

**Fixed effects**	**Estimate**	**Std. Error**	***z* value**	***P* value**
Species	-0.046	0.487	-0.095	0.924
Condition	0.999	0.306	3.263	0.001 **
Species * Condition	0.147	0.429	0.342	0.733

Note. Significance codes: '***' < 0.001; '**' < 0.01; '*' < 0.05; '.' < 0.1.

Table S3 *P* values and related parameters for the main effects and their interaction on Body-touch frequency (GLMM)

Contrast	Estimate	SE	df	z ratio	*P* value
Dog, Control - Pig, Control	0.046	0.487	Inf	0.095	0.9997
Dog, Control - Dog, Food	-0.999	0.306	Inf	-3.263	0.006 **
Pig, Control - Pig, Food	-1.145	0.301	Inf	-3.806	0.0008 ***
Dog, Food - Pig, Food	-0.1	0.384	Inf	-0.262	0.994

Note. Results are given on the log (not the response) scale. Significance codes: '***' < 0.001; '**' < 0.01; '*' < 0.05; '.' < 0.1. Tukey method for *P* value adjustment

Table S4 *P* values and related parameters of the post hoc tests for the contrasts on Body-touch frequency

Fixed effects	Estimate	Std. Error	*z* value	*P* value
Species	-1.601	0.519	-3.083	0.002 **
Condition	1.099	0.215	5.1	<0.0001 ***
Species * Condition	0.916	0.478	1.915	0.055 .

Note. Significance codes: '***' < 0.001; '**' < 0.01; '*' < 0.05; '.' < 0.1.

Table S5 P values and related parameters for the main effects and their interaction on Face-orientation frequency (GLMM)

Contrast	Estimate	SE	df	z ratio	*P* value
Dog, Control - Pig, Control	1.601	0.519	Inf	3.083	0.011 *
Dog, Control - Dog, Food	-1.099	0.215	Inf	-5.1	<0.0001***
Pig, Control - Pig, Food	-2.015	0.427	Inf	-4.717	<0.0001***
Dog, Food - Pig, Food	0.684	0.327	Inf	2.093	0.155

Note. Results are given on the log (not the response) scale. Significance codes: '***' < 0.001; '**' < 0.01; '*' < 0.05; '.' < 0.1. Tukey method for *P* value adjustment.

Table S6. *P* values and related parameters of the post hoc tests for the contrasts on Face-orientation frequency

Supplementary Figure

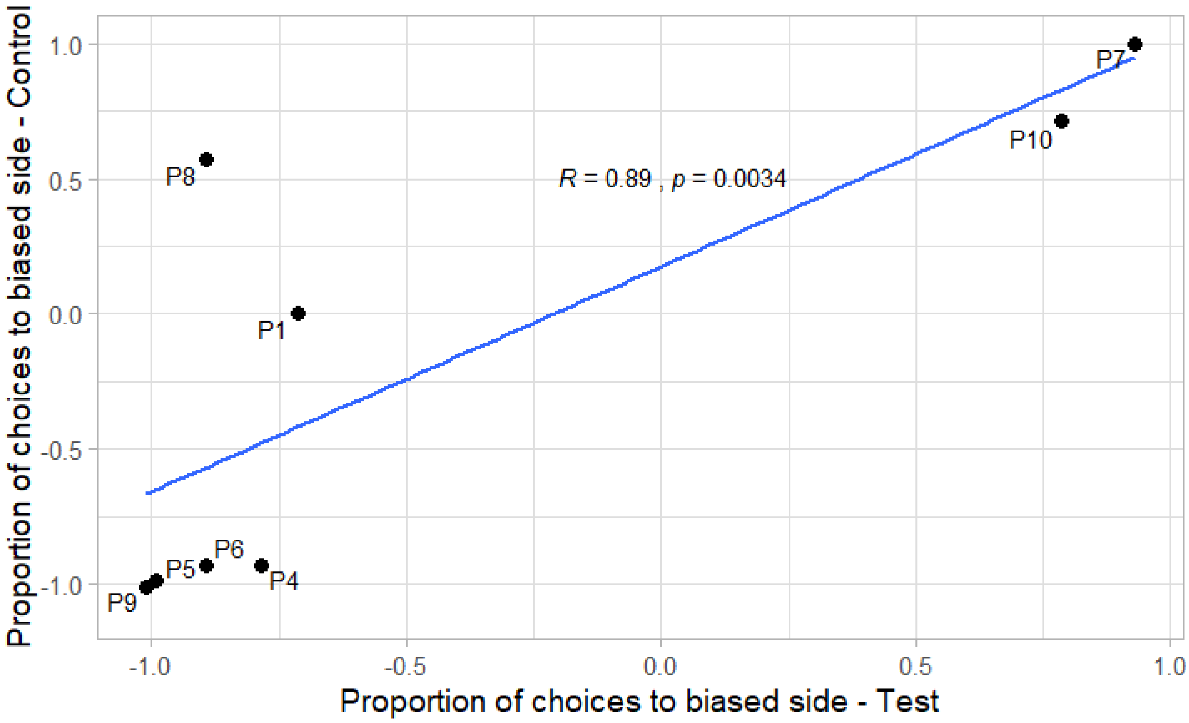

Fig. S1 The proportion of choices to the biased side for each pig individual the in the test and control trials. Negative values show bias to the left, while positive values show bias to the right side. Data point labels stand for the individuals (corresponding to those on Fig. 7).
